# Lifestyle Behaviours, Self-Esteem and Academic Performance in Primary Education Students—A Structural Equation Model According to Sex and School Type

**DOI:** 10.3390/children10111769

**Published:** 2023-10-31

**Authors:** Gracia Cristina Villodres, Federico Salvador-Pérez, Ramón Chacón-Cuberos, José Joaquín Muros

**Affiliations:** 1 Department of Didactics of Corporal Expression, Faculty of Education, University of Granada, 18071 Granada, Spain; gcvillodres@ugr.es; 2 Department of Didactics of Social Sciences, Faculty of Education, International University of La Rioja, 26006 Logroño, Spain; federico.salvador@unir.net; 3 Department of Research Methods and Diagnosis in Education, Faculty of Education, University of Granada, 18071 Granada, Spain; rchacon@ugr.es

**Keywords:** physical activity, Mediterranean diet adherence, screen time, maximal oxygen uptake, self-esteem, academic performance, children, preadolescence, structural equation model

## Abstract

(1) Background: The present study aimed to examine the relationship between physical activity (PA), screen time (ST), maximal oxygen uptake (VO_2_max), Mediterranean diet (MD) adherence, self-esteem (SE) and academic performance (AP) in primary education students. In order to address this aim, an explanatory model was developed to examine the existing relationships between PA, ST, VO_2_max, MD adherence, SE and AP. Further, the proposed structural model was examined via multi-group analysis as a function of sex and school type. (2) Methods: A non-experimental, descriptive, comparative and cross-sectional study was designed with a total sample of 269 Spanish students (11.29 ± 0.62). Validated questionnaires were administered to collect data on study variables. (3) Results: Relative to boys, girls reported better academic grades and showed a stronger positive relationship between MD adherence and AP, MD adherence and PA, and VO_2_max and SE. Likewise, girls showed a stronger negative relationship between ST and VO_2_max, and ST and MD adherence. At the same time, mixed funding school (MFS) students reported higher PA engagement than state school (SS) students. However, SS students reported better MD adherence, ST and AP than MFS students. Further, a stronger positive relationship was found in SS students between MD adherence or VO_2_max and SE than in MFS students. Also, within the former group, ST was more negatively related to MD adherence and VO_2_max. (4) Conclusions: Scientific and educational communities must develop future strategies that consider potential determinants in order to target more desirable outcomes.

## 1. Introduction

Pre-adolescence is an important developmental period characterised by the establishment of behavioural habits which can affect children’s health both now and in the future [1]. 

Physical activity (PA) has been defined as any exercise performed using the skeletal muscles and involving energetic expenditure [2]. According to World Health Organization (WHO) recommendations, children and adolescents should engage in at least 60 min of moderate-to-vigorous PA per day. Further, it is recommended not to spend more than two hours per day in front of screens or devices [3]. Overwhelming evidence associates PA with a reduction in cardiovascular diseases, hypertension, different cancers, etc. [4]. Also, PA engagement translates into physical and mental benefits—such as improved maximal oxygen uptake (VO_2_max), self-esteem (SE) [5,6], cognitive functioning and academic performance (AP) [7].

In the majority of scientific studies, children reporting greater PA engagement also report adequate Mediterranean diet (MD) adherence and less screen time (ST) [8]. In addition to PA engagement, greater cardiorespiratory fitness is associated with optimal MD adherence in children aged between six and 13 years [9]. MD is characterised by a low intake of saturated fats and red meat and a limited amount of wine in adults, while emphasising a high consumption of plant-based foods like fruits, vegetables, whole grains, nuts and seeds. A MD also includes olive oil, dairy products such as cheese and yogurt, eggs, and a moderate consumption of fish and poultry [10]. It is characterised by low levels of saturated fats and elevated levels of monounsaturated fats, alongside abundant fibre, complex carbohydrates and antioxidants [11]. It is also known that following a traditional MD helps to maintain a healthy body weight [12] and general health-related quality of life in children and adolescents [13]. Further, poor MD adherence in children is associated with a higher risk of suffering cognitive issues and mental illnesses such as attention deficit hyperactivity disorder (ADHD) [14,15]

Previous research also highlights that health promotion interventions targeting physical fitness and sound nutrition in children benefit not only physical and mental health, such as SE, but also cognitive health [16], such as AP [17]. Black et al. [18] observed that a multiple micronutrient-fortified diet increased school children’s cognition with improved expressive language and inhibitory control. At the same time, Ludyga et al. [19] found that a PA programme run during school break time improved adolescents’ executive functions, such as inhibitory control. Likewise, a metanalysis conducted by Rasberry et al. [20] revealed a total of 251 associations between PA and AP, with AP being represented through measures of academic performance, cognitive skills, attitudes and academic grades in Mathematics.

Although physical and mental health benefits are widely acknowledged, approximately 81% of adolescents aged 11–17 do not engage in enough PA. Among this group, girls are less active than boys, with 85% of girls failing to meet recommendations set by the WHO compared to 78% of boys [2]. Also, Spanish children and adolescents spend one hour and 13 min a day on weekdays and almost three hours a day on weekend days in front of a screen, exceeding screen recommendations, with ST being higher in boys [3]. According to the latest Pasos study [3], more than 12% of the Spanish population aged eight to sixteen years reported low MD adherence. Similar to the results seen with PA engagement, girls reported lower MD adherence than boys.

Further, the present research team observed that confinement due to COVID-19 caused PA to decrease in Spanish children, whilst ST increased, negatively affecting SE. In this regard, it is essential to consider SE from early ages in order to improve health-related quality of life [21]. Also, SE is considered to be a main criterion for the diagnosis of mental disorders [22]. Specifically, girls and state school (SS) students were most affected during the pandemic [23]. Concern exists that these habits will continue into the future [24]. 

In line with that discussed above, Schlund et al. [25] concluded that sex effects are not reported in a suitable way for effective intervention programmes to be developed to improve healthy habits at school. A consideration of sex is imperative as it is key at every step of intervention planning, implementation and evaluation. Likewise, according to Muñiz and Suárez-Pandiello [26], students’ immediate settings, such as their school, can influence their health. In Spain, broad differences exist between mixed funding schools (MFS) and SS. Typically, students attending MFS tend to be predominantly Catholic and come from families with a higher socioeconomic status than SS students.

The present study hypothesises that sex and setting could be influential factors in the health of children. Consequently, influential factors in the aforementioned relationships need to be considered in order to provide a scientific basis from which future intervention programmes targeting healthy habits can be developed. It is important to examine whether differences emerge as a function of sex and setting. 

Thus, the present study aimed to examine the relationship between PA, ST, VO_2_max, MD adherence, SE and AP in primary education students. In order to address this aim, the present study outlined the following objectives:

Develop an explanatory model of the existing relationships between PA, ST, VO_2_max, MD adherence, SE and AP in primary education students;

Analyse the associations between the variables included in the model according to sex and school type through multi-group analysis.

## 2. Materials and Methods

### 2.1. Design and Participants

A non-experimental, descriptive, comparative and cross-sectional study was designed.

Three hundred and thirty-one children were initially recruited to the study. Of these, questionnaires completed by 62 participants were discarded due to incorrect completion or missing data. Thus, 269 students formed the final sample. The sample was made up of 125 girls (46.50%) and 144 boys (53.50%), with an average age of 11.29 ± 0.62 years. All participants came from two state schools (SS) and three mixed-funding schools (MFS) in Granada, Spain.

The sampling process was non-random, as participants were chosen based on convenience. All students willingly took part in the study, with the prior consent of their parents or legal guardians. The study was authorised by participating schools and parents’ associations.

### 2.2. Instruments

A Spanish version of the PA questionnaire for children (PAQ-C) that has been cross-culturally adapted and validated in children aged between eight and fourteen years was used to evaluate PA engagement [27,28]. This tool has been shown to be valid and reliable, producing an intraclass correlation coefficient (ICC) greater than 0.73 and an internal consistency of α = 0.86. This self-report instrument comprises 10 questions aimed at gauging involvement in moderate and vigorous PA over the week prior to completion. An overall PA score is determined by calculating average scores from responses to the first nine questions, which are rated on a five-point scale. The tenth question serves as a validity check, in which respondents indicate whether any personal obstacles hindered their regular PA during the seven days evaluated by the questionnaire. None of the respondents mentioned facing such hindrances.

VO_2_max was estimated using the 20 m incremental maximum effort shuttle run field test, employing the equation proposed by Léger et al. [29]. In order to complete this test, participants must run back and forth between two lines placed 20 m apart. Participants start at an initial velocity of 8.5 km/h and increase their speed by 0.5 km/h/min. When participants can no longer reach the line within the time provided on two consecutive occasions or can no longer maintain the physical effort required to continue, they stop and finish the test. 

The latest updated version by Altavilla et al. [30] of the KIDMED [31] was used to evaluate MD adherence. This test consists of 16 yes-or-no items that evaluate MD adherence in children and adolescents. Twelve questions are phrased positively, where a ‘yes’ response is scored as +1. Conversely, four questions are phrased negatively, where a ‘yes’ response is scored as −1. All ‘no’ responses are scored as 0. The maximum possible score is 12, whilst the lowest possible score is −4. Based on the total KIDMED score, children’s diets are categorised as either optimal quality (≥8), needing improvement (4–7) or poor quality (≤3).

An ad hoc and bespoke questionnaire was developed to collect the sociodemographic data of sex and date of birth. Also, ST was evaluated. Children reported the number of hours spent daily on screen-based leisure activities such as watching television, playing video games, using a mobile phone, using a computer, etc., on weekdays and weekends. An overall summary score was calculated from the mean number of hours reported over the seven days examined (week and weekend days).

AP was evaluated according to academic grades. Schools provided recorded grades for nine different subjects: Natural Sciences, Social Sciences, Spanish Language and Literature, Mathematics, English, Religion/Values, Art Education, Physical Education and French. Grades for these subjects pertained to the first term of the school year in which the research was carried out (2022–2023). Previous studies have also used these indicators to assess AP [7].

An adapted, translated and validated Spanish version of the Rosenberg self-esteem scale was used for the evaluation of personal SE [32,33]. This adapted version has demonstrated both validity and reliability, with an internal consistency of α = 0.80. The scale comprises ten items designed to gauge an individual’s SE levels. The first five items are phrased positively, with responses graded from A to D and assigned scores ranging from 4 to 1. The remaining five items are negatively phrased, and for these questions, responses are listed from A to D, with scores ranging from 1 to 4. This design helps account for potential acquiescence bias in self-administered assessments. Total scores are computed by summing scores for each specific item, resulting in a maximum possible score of 40 and a minimum score of 10. Final scores are categorised as follows: low SE, indicating significant SE issues (10–25); medium SE, suggesting room for improvement (26–29); and high SE, reflecting a normal level of SE (30–40).

The Spanish translation of the most recent version of the family affluence scale (FAS III) was used to evaluate social economic status (SES) [34,35]. This scale has been shown to be valid and reliable, with an internal consistency of between α= 0.76 and 0.91. This scale consists of six items that are designed to evaluate family purchasing power based on material goods. All items were scored between 0 and 3. The sum of individual scores was used to evaluate total SES. The highest possible score is 13 and the lowest possible score is 0 [36]. SES was classified as low (0–2), medium (3–5) or high (≥6). In this case, all participants showed a high socioeconomic level.

### 2.3. Procedure

Two sets of informational packages were developed to ensure informed consent. The first was directed towards educational centre directors, while the second was intended for the parents or legal guardians of participating students. These packages contained comprehensive details regarding the study’s attributes and prerequisites. Furthermore, a research assistant was available to offer assistance and guidance regarding the performance of physical testing.

Participants were instructed on the correct completion of questionnaires and tests. All tests were conducted during school time. Ethical approval was granted by the Ethics Committee of the University of Granada (2796/CEIH/2022). 

Data collection was carried out during the months of April and May 2023.

### 2.4. Data Analysis

Data were analysed using IBM SPSS 25.0 statistical software. Categorical data such as sex, school year and school type are represented as percentages. For numerical data, means and standard deviations are presented. Sample distribution was evaluated using the Kolmogorov–Smirnov test. Upon confirming a non-normal distribution, the Mann–Whitney U test was employed to compare two independent groups and the Kruskal–Wallis test was used to compare more than two groups.

**Figure 1:**
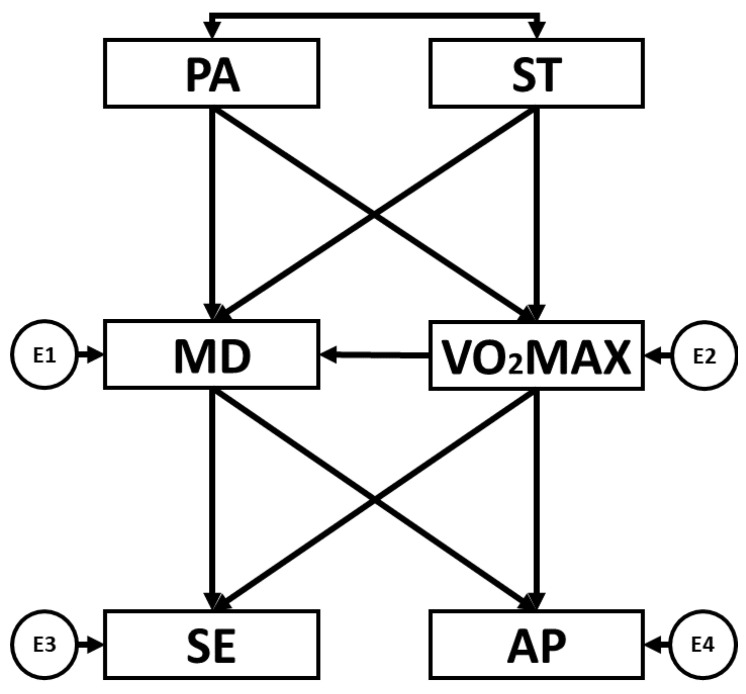
Structural equation model. Note: physical activity (PA), screen time (ST), Mediterranean diet (MD), maximal oxygen uptake (VO_2_max), self-esteem (SE), academic performance (AP).

IBM AMOS^®^ 24.0 software was used to perform structural equation modelling (SEM). SEM enables connections to be established between the variables outlined in the theoretical model (Figure 1). In this instance, the model was constructed with six observed variables. These observed variables included the four endogenous variables, SE, AP, MD adherence and VO_2_max, and the two exogenous variables, PA and ST. Endogenous variables have associated error terms which are visually depicted by a circle, whilst exogenous variables lack error terms and are represented by two-way arrows. SEM was employed to determine the relationships between the variables in the theoretical model as a function of sex (boys and girls) and school type (SS and MFS).

In order to evaluate the fit of the developed structural equations model (SEM) with the actual data, various indices were used to evaluate model adequacy. The goodness of fit should be assessed on the basis of a chi-square test. Non-significant *p*-values indicate a good model fit. According to Byrne [37], it is also important to use additional fit indices because the mentioned statistic can be highly influenced by the sample size. Comparative fit index (CFI), incremental fit (IFI), normalised fit (NFI) and the Tucker–Lewis (TLI) indices were used. In order to be able to conclude that model fit is acceptable, values should exceed 0.90, with excellent model fit being indicated by values higher than 0.95. In addition, root mean squared error of approximation (RMSEA) analysis was performed, with values below 0.08 signifying acceptable fit and values below 0.05 indicating excellent fit.

## 3. Results 

Descriptive characteristics of the study sample are presented in Table 1.

Of the total number of students, 53.5% were boys and 46.5% were girls. Boys reported significantly higher PA engagement (3.65 ± 0.90 vs. 3.29 ± 0.82; *p* < 0.001) and VO_2_max than girls (41.82 ± 6.37 vs. 37.45 ± 5.74 mL/kg/min; *p* < 0.001). However, girls achieved significantly higher academic grades (8.15 ± 1.16 vs. 7.77 ± 1.22; *p* = 0.014) than boys.

No significant differences were found with regard to ST, MD adherence and SE.

In addition, 42.4% attended SS, whilst 57.6% of participating students attended MFS. Statistically significant differences were observed in relation to ST, with this being higher in MFS students (2.68 ± 1.46 vs. 1.82 ± 1.51 h; *p* < 0.001). Likewise, students belonging to MFS reported higher PA engagement (3.72 ± 0.82 vs. 3.17 ± 0.85; *p* < 0.001). However, SS students reported higher MD adherence (7.12 ± 2.51 vs. 5.92 ± 2.8; *p* < 0.001) and AP (8.21 ± 1.20 vs. 7.75 ± 1.8; *p* < 0.001) than MFS students.

No significant differences were found with regard to VO_2_max and SE.

Important differences are revealed according to sex and school type. For this reason, a structural equation model was carried out in order to better understand the relationship between study variables and the influence of student groupings on outcomes.

The structural model developed showed good fit indices for the multi-group analysis. The chi-squared test produced statistically significant outcomes (χ^2^ = 4.1; df = 5; *p* = 0.539). This meant that the null hypothesis was accepted. The model could also be concluded to be homogeneous. However, Byrne (2016) urges the need to use other goodness of fit indices, given the sensitivity to sample size that is presented by this statistic. In this sense, NFI, IFI, TLI and CFI values were all excellent, being 0.97, 1.007, 1.023 and 1.00, respectively. Likewise, the RMSEA value was 0.000, which was also excellent. Thus, an excellent fit was demonstrated between the SEM developed and the gathered empirical data.

**Figure 2:**
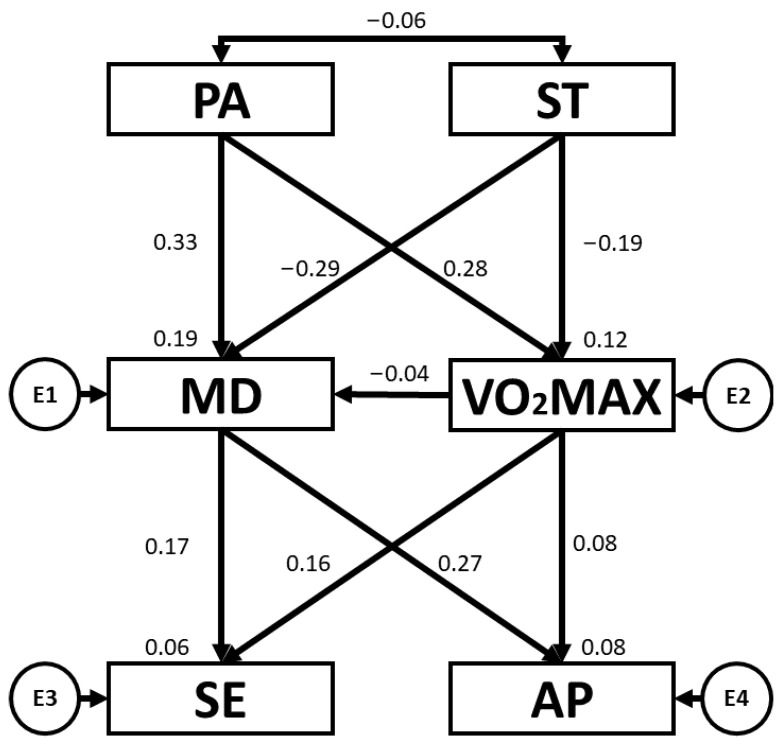
Structural equation model for the overall sample. Note: physical activity (PA), screen time (ST), Mediterranean diet (MD), maximal oxygen uptake (VO_2_max), self-esteem (SE), academic performance (AP).

Table 2 and Figure 2 present the regression weights and standardised regression weights pertaining to the SEM developed for the overall sample. These outcomes make it possible to determine existing associations between PA, ST, VO_2_max, MD adherence, SE and AP. Positive relationships were observed between PA and VO_2_max (b = 0.278; *p* < 0.005) and PA and MD adherence (b = 0.325; *p* < 0.005). With regard to ST, negative relationships were observed between ST and VO_2_max (b = −0.188; *p* < 0.001) and ST and MD adherence (b = −0.288; *p* < 0.005). MD adherence was positively related to AP (b = 0.272; *p* < 0.005) and SE (b = 0.173; *p* = 0.004). Likewise, VO_2_max was positively related to SE (b = 0.162; *p* = 0.006).

**Figure 3:**
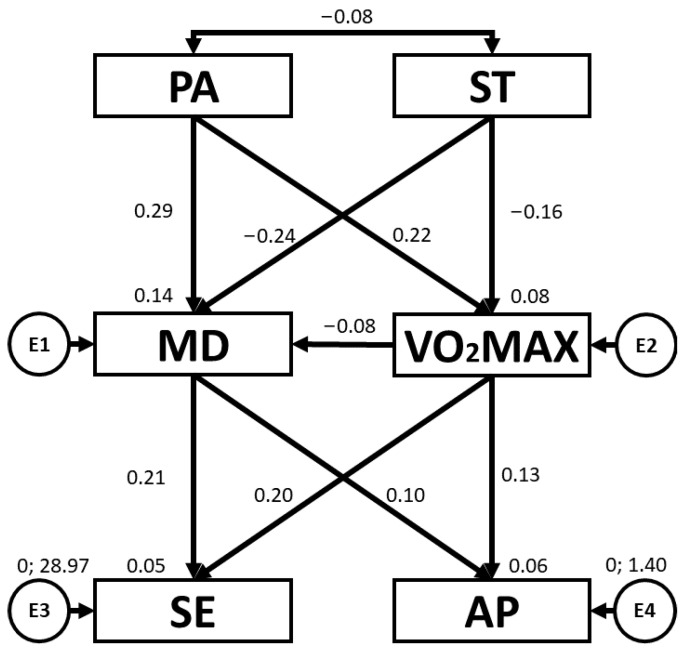
Structural equation model for boys. Note: physical activity (PA), screen time (ST), Mediterranean diet (MD), maximal oxygen uptake (VO_2_max), self-esteem (SE), academic performance (AP).

Table 3 and Figure 3 present the regression weights and standardised regression weights pertaining to the SEM developed for boys. The chi-squared test produced a statistically significant outcome (χ^2^ = 2.0; df = 5; *p* = 0.844). As before, the NFI value obtained was 0.962, the IFI value was 1.061, the TLI value was 1.228 and the CFI value was 1.00, with all of these being excellent. Likewise, the RMSEA obtained a value of 0.000, which was also excellent and demonstrated an appropriate level of fit of the SEM. In this case, PA was positively related to VO_2_max (b = 0.222; *p* = 0.006) and MD adherence (b = 0.291; *p* < 0.005). With regard to ST, negative relationships were observed between ST and VO_2_max (b = −0.160; *p* = 0.046) and ST and MD adherence (b = −0.244; *p* = 0.002). In addition, MD adherence was positively related to AP (b = 0.202; *p* = 0.013) and SE (b = 0.206; *p* = 0.011).

**Figure 4:**
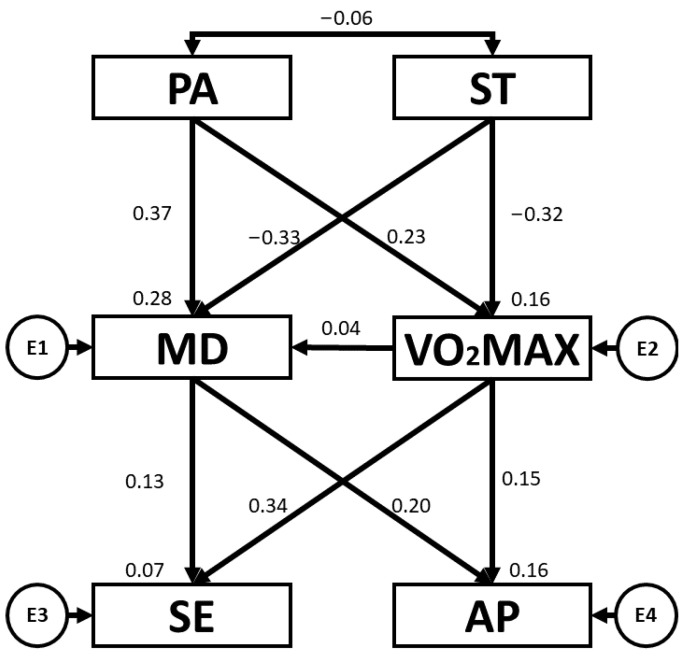
Structural equation model for girls. Note: physical activity (PA), screen time (ST), Mediterranean diet (MD), maximal oxygen uptake (VO_2_max), self-esteem (SE), academic performance (AP).

Table 4 and Figure 4 present the regression weights and standardised regression weights pertaining to the SEM developed for girls. The chi-squared test produced a statistically significant outcome (χ^2^ = 2.4; df = 5; *p* = 0.794). As before, the NFI value obtained was 0.975, the IFI value was 1.029, the TLI value was 1.097 and the CFI value was 1.00, with all of these being excellent. Likewise, the RMSEA obtained a value of 0.000, which was also excellent and demonstrated an appropriate level of fit of the SEM. In this case, PA was positively related to VO_2_max (b = 0.226; *p* = 0.006) and MD adherence (b = 0.370; *p* < 0.005). With regard to ST, negative relationships were observed between ST and VO_2_max (b = −0.318; *p* < 0.005), and ST and MD adherence (b = −0.329; *p* < 0.005). MD adherence was positively related to AP (b = 0.342; *p* < 0.005). Also, a positive relationship was observed between VO_2_max and SE (b = 0.204; *p* = 0.022).

**Figure 5:**
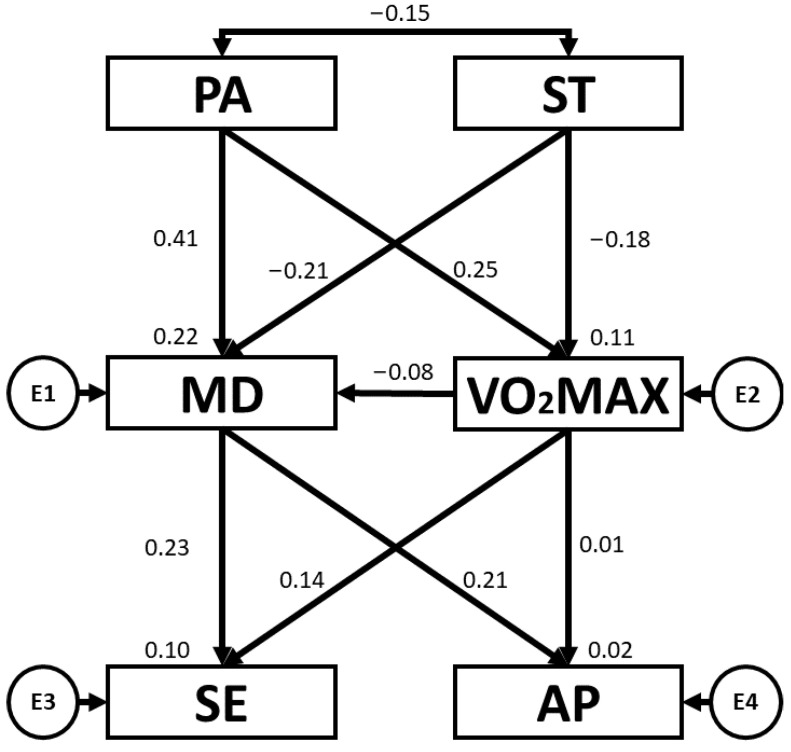
Structural equation model for participants attending state schools. Note: physical activity (PA), screen time (ST), Mediterranean diet (MD), maximal oxygen uptake (VO_2_max), self-esteem (SE), academic performance (AP).

Table 5 and Figure 5 present the regression weights and standardised regression weights pertaining to the SEM developed for participants attending SS. The outcome of the chi-squared test was statistically significant (χ^2^ = 1.5; df = 5; *p* = 0.918). As before, the NFI value obtained was 0.975, the IFI value was 1.066, the TLI value was 1.242 and the CFI value was 1.00, with all of these being excellent. Likewise, the RMSEA obtained a value of 0.000, which was also excellent and demonstrated an appropriate level of fit of the SEM. In this case, PA was positively related to VO_2_max (b = 0.247; *p* = 0.006) and MD adherence (b = 0.405; *p* < 0.005). With regard to ST, negative relationships were observed between ST and VO_2_max (b = −0.181; *p* = 0.044) and ST and MD adherence (b = −0.211; *p* = 0.014). MD adherence was positively related to SE (b = 0.231; *p* = 0.010). Also, a positive relationship was observed between VO_2_max and SE (b = 0.209; *p* = 0.019).

**Figure 6:**
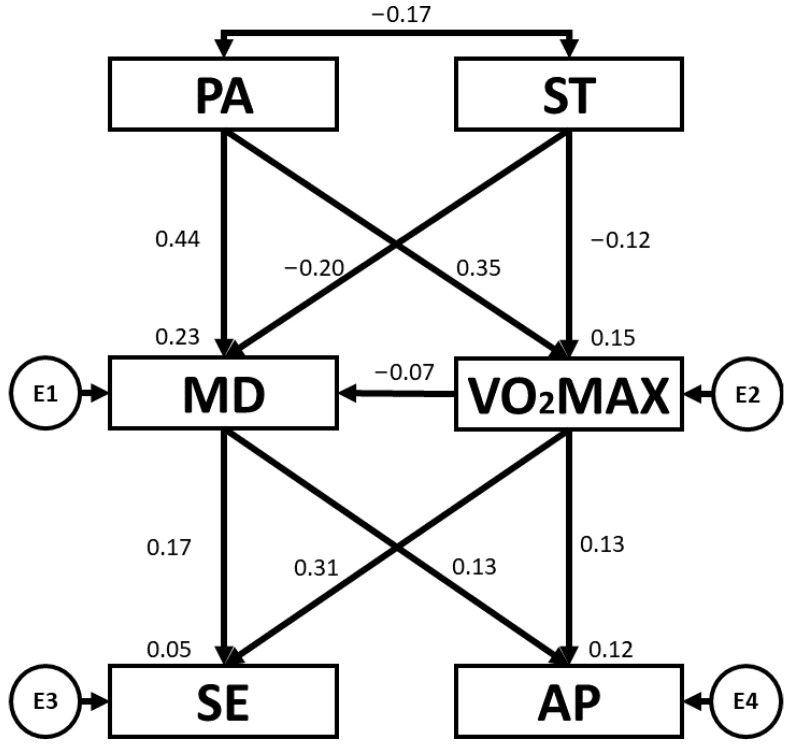
Structural equation model for participants attending mixed funding schools. Note: physical activity (PA), screen time (ST), Mediterranean diet (MD), maximal oxygen uptake (VO_2_max), self-esteem (SE), academic performance (AP).

Table 6 and Figure 6 present the regression weights and standardised regression weights pertaining to the SEM developed for participants attending MFS. The chi-squared test produced a statistically significant outcome (χ^2^ = 4.6; df = 5; *p* = 0.470). As before, the NFI value obtained was 0.955, the IFI value was 1.004, the TLI value was 1.014 and the CFI value was 1.00, with all of these being excellent. Likewise, the RMSEA obtained a value of 0.000, which was also excellent and demonstrated an appropriate level of fit of the SEM. In this case, PA was positively related to VO_2_max (b = 0.348; *p* < 0.005) and MD adherence (b = 0.435; *p* < 0.005). With regard to ST, a negative relationship was observed between ST and MD adherence (b = −0.195; *p* = 0.007). In addition, MD adherence was positively related to AP (b = 0.310; *p* < 0.005) and SE (b = 0.171; *p* = 0.031).

## 4. Discussion

Preadolescence is a key period for the development of physical, mental and cognitive health. The first aim of the present research was to define an explanatory model of the existing relationships between PA, ST, VO_2_max, MD adherence, SE and AP in primary education students. The second aim was to analyse the associations between the variables included in the model according to sex and school type through a multi-group analysis. A good fit was obtained for the proposed models.

The findings of the present study established that preadolescent boys exhibited higher PA engagement and VO_2_max scores than preadolescent girls. Similar findings have been reported by the majority of existing research on the topic and the WHO [2]. Indeed, previous Spanish research has argued that access barriers cause women to engage in alarmingly less PA than men [38].

The present study presents potentially contentious finding with regard to the positive relationship between PA and AP found in previous studies [7]. In this case, girls are less active than boys; however, girls reported better academic grades than boys. Nowadays, such controversial outcomes are subject for discussion. A recent review of over 100 randomised controlled trials [39] investigating the effects of regular PA on cognition in childhood and adolescence showed that available evidence does not allow definitive conclusions regarding the cognitive benefits of PA.

In the same way, a stronger positive relationship was found in girls between MD adherence and AP than in boys. Further, despite SS students being less active than MFS students, the former achieved better academic grades. In addition, SS students showed greater MD adherence. This suggests that research should place greater emphasis on examining the relationship between MD adherence and AP. Esteban-Cornejo et al. [40] concluded that MD adherence was related to AP after adjusting for confounders. Specifically, individuals with higher MD adherence reported higher scores for all examined academic indicators than individuals with low MD adherence. Accordingly, the Health Behaviour in School-Aged Children (HBSC) study [15] observed that following a poor diet can cause metabolic changes and negatively affect physical, cognitive and emotional performance. With regard to cognitive problems, lack of concentration, impaired speech or expression capacity, memory, creativity and problem solving stand out [15]. Various nutrients and specific foods have displayed inconsistent correlations with cognitive function in adults. This previous research considered certain vitamins, carotenoids, long-chain n-3 Polyunsaturated Fatty Acids (PUFAs) found in seafood and whole foods abundant in polyphenols like fruits, vegetables, nuts, olive oil and coffee [41,42,43]. However, such detailed analyses are lacking in children.

Further, a stronger positive relationship was found between PA and MD adherence and between VO_2_max and SE in girls than in boys. At the same time, the negative relationship between VO_2_max and ST was more pronounced in girls. Bergier et al. [44] observed that girls typically engage in better nutritional habits because they are more concerned with losing weight, which is connected with one of the most substantial problems in the contemporary world. A potential explanation for this is that girls tend to adopt healthy behaviours (PA engagement, improved VO_2_max and MD adherence) in order to achieve a better figure, with concomitant effects on SE, whilst boys tend to engage in PA for personal enjoyment. 

At the same time, MD adherence and VO_2_max were found to be more strongly related to SE in SS students than in MFS students, with MD adherence and VO_2_max being more strongly negatively related to ST in the former student group. Castro et al. [45] observed that SS students were more likely than mixed funding or private school students to adhere to recommendations regarding the monthly frequency of meals. This could suggest that SS students are more likely to engage in healthful behaviour, in general, with this having a positive impact on their SE that is not felt by MFS students.

Likewise, the present study found that SS students reported lower PA engagement. Despite this, these students reported higher ST than MFS students. This could suggest that MFS students may receive a higher quality education, with this influencing their health habits. However, Muñiz and Suárez-Pandiello [26] reported that, after controlling for SES, both types of schools obtained similar academic outcomes. Thus, in striving to understand inconsistencies in findings, a study conducted by Constandst et al. [46] was considered which concluded that PA engagement may be encouraged through the use of electronic devices during confinement due to COVID-19. Such habits may have prevailed following confinement. Further, previous research has determined that Spanish parents from higher socioeconomic backgrounds are more inclined to enrol their children in MFS [47], given that they can afford the fees, enabling their children to bypass selective admission procedures. In this sense, MFS students have greater access to technological devices to engage not only in sedentary activities, such as playing video games, but also in PA through the use of social networks such as YouTube or online coaching.

### Limitations and Future Perspectives

The present study has some limitations that require consideration. Suggestions are presented to enhance the reliability and reach of future research.

Firstly, this study relied on questionnaires to measure the study variables, introducing a potential risk of measurement error due to recall and social desirability bias. Nevertheless, the tools utilised (PAQ-C, KIDMED, Course Navette, Rosenberg scale) have demonstrated adequate reliability and validity for application in this population. Thus, the anticipated impact on the final conclusions is minimal. Alternatively, future research could employ accessible and convenient technological devices like pulsometers or accelerometers, which provide more precise data regarding the frequency and intensity of PA at specific time intervals.

Also, sleep quality could be a confounding variable that may influence the relationship between examined variables [48] but which could not be evaluated here. Likewise, a direct measure of heart rate response during the 20 m incremental shuttle run test (Course Navette test) could be a relevant indicator of an individual’s physical fitness level [49]. Both should be considered in future studies.

Furthermore, the present study was cross-sectional, involving a single assessment within a particular population at a specific point in time. This approach only permits the identification of associations between variables at the time of examination, precluding the establishment of causal relationships. It would be beneficial to conduct longitudinal studies or experimental designs to determine the causal effects of studied variables. 

Moreover, the sample was drawn from a highly specific geographic region, limiting the generalisability of findings to broader geographic areas, be it at a national or regional scale. It would be valuable to replicate the study in different populations and educational settings to determine whether similar relationships exist.

In terms of future perspectives, it would be valuable to design, implement and evaluate the impact of educational materials that promote the adoption of the healthful behaviours under scrutiny. These resources should particularly target the most susceptible demographic groups, such as young girls or individuals from socioeconomically disadvantaged backgrounds.

## 5. Conclusions

The findings of the present study are in line with those reported in the previously published literature. A positive correlation was observed between PA and VO_2_max, PA and MD adherence, and VO_2_max and SE. In contrast, ST was negatively correlated with VO_2_max and MD adherence. These results are in line with those reported in the available literature. Further, social barriers continue to affect girls, who reported lower PA engagement and had poorer VO_2_max scores than boys. 

Despite this, girls reported better academic grades than boys. At the same time, a stronger positive relationship emerged between MD adherence and AP, MD adherence and PA, and VO_2_max and SE in girls than in boys. Likewise, a stronger negative relationship emerged between ST and VO_2_max, and ST and MD adherence in girls. Research should improve knowledge about the relationship between PA engagement and MD adherence and cognition in order to draw more solid conclusions. In addition, schools must seek to increase the motivation of girls to engage in sport for personal enjoyment, as opposed to as a means of improving body image. 

At the same time, MFS students reported higher PA engagement than SS students. However, SS students reported better MD adherence, ST and AP than MFS students. Further, a stronger positive relationship emerged between MD adherence and VO_2_max and SE in SS students than in MFS students. Further, a stronger negative relationship emerged between MD adherence and VO_2_max and ST within the former group. Adherence to nutritional recommendations outlined by school canteens and family SES may be influential factors determining healthy habits in students.

In summary, scientific and educational communities must study, outline and develop future strategies that consider the potential determinants discussed throughout the present study in order to target more desirable outcomes. 

## Figures and Tables

**Table 1:** Sample characteristics according to sex and school type.

Characteristics		n	%	PA	*p*	ST	*p*	MD	*p*	VO_2_max	*p*	SE	*p*	AP	*p*
Total		269	100	3.49 ± 0.88		2.31 ± 1.54		6.43 ± 2.74		39.79 ± 6.46		30.48 ± 5.99		7.85 ± 1.21	
Sex	Boys	144	53.5	3.65 ± 0.90	<0.001	2.39 ± 1.66	0.628	6.41 ± 2.74	0.820	41.82 ± 6.37	<0.001	30.94 ± 5.55	0.280	7.77 ± 1.22	<0.001
	Girls	125	46.5	3.29 ± 0.82		2.21 ± 1.39		6.43 ± 2.74		37.45 ± 5.74		29.94 ± 6.43		8.15 ± 1.16	
School Type	SS	114	42.4	3.17 ± 0.85	<0.001	1.82 ± 1.51	<0.001	7.12 ± 2.51	<0.001	40.27 ± 6.73	0.684	30.04 ± 6.83	0.733	8.21 ± 1.20	<0.001
	MFS	155	57.6	3.72 ± 0.82		2.68 ± 1.46		5.92 ± 2.8		39.43 ± 6.51		30.81 ± 5.28		7.75 ± 1.8	

Note: physical activity (PA), screen time (ST), Mediterranean diet (MD), maximal oxygen uptake (VO_2_max), self-esteem (SE), academic performance (AP), state school (SS), mixed funding school (MFS).

**Table 2:** Regression weights for the overall sample.

Association between Variables			RW				SRW
			Estimation	SE	CR	*p*	Estimation
VO_2_max	←	PA	2.043	0.423	4.831	***	0.278
VO_2_max	←	ST	−0.788	0.240	−3.276	0.001	−0.188
MD	←	PA	1.016	0.180	5.655	***	0.325
MD	←	VO_2_max	−0.016	0.025	−0.642	0.521	−0.038
MD	←	ST	−0.512	0.100	−5.118	***	−0.288
AP	←	MD	0.120	0.026	4.632	***	0.272
SE	←	MD	0.376	0.130	2.898	0.004	0.173
SE	←	VO_2_max	0.150	0.055	2.726	0.006	0.162
AP	←	VO_2_max	0.014	0.011	1.290	0.197	0.076
PA	↔	ST	−0.080	0.082	−0.976	0.329	−0.060

Note: regression weight (RW), standardised regression weight (SRW), standard error (SE), critical ratio (CR), maximal oxygen uptake (VO_2_max), physical activity (PA), screen time (ST), Mediterranean diet (MD), self-esteem (SE), academic performance (AP). *** *p* < 0.005.

**Table 3:** Regression weights pertaining to the SEM developed for boys.

Association between Variables			RW				SRW
			Estimation	SE	CR	*p*	Estimation
VO_2_max	←	PA	1.582	0.573	2.762	0.006	0.222
VO_2_max	←	ST	−0.615	0.308	−1.992	0.046	−0.160
MD	←	PA	0.889	0.243	3.653	***	0.291
MD	←	VO_2_max	−0.033	0.035	−0.939	0.348	−0.076
MD	←	ST	−0.402	0.130	−3.105	0.002	−0.244
AP	←	MD	0.090	0.036	2.490	0.013	0.202
SE	←	MD	0.418	0.165	2.531	0.011	0.206
SE	←	VO_2_max	0.083	0.071	1.176	0.240	0.096
AP	←	VO_2_max	0.025	0.016	1.581	0.114	0.128
PA	↔	ST	−0.123	0.124	−0.990	0.322	−0.083

Note: regression weight (RW), standardised regression weight (SRW), standard error (SE), critical ratio (CR), maximal oxygen uptake (VO_2_max), physical activity (PA), screen time (ST), Mediterranean diet (MD), self-esteem (SE), academic performance (AP). *** *p* < 0.005.

**Table 4:** Regression weights pertaining to the SEM developed for girls.

Association between Variables			RW				SRW
			Estimation	SE	CR	*p*	Estimation
VO_2_max	←	PA	1.588	0.578	2.747	0.006	0.226
VO_2_max	←	ST	−1.313	0.340	−3.861	***	−0.318
MD	←	PA	1.248	0.266	4.688	***	0.370
MD	←	VO_2_max	0.018	0.040	0.450	0.653	0.038
MD	←	ST	−0.653	0.161	−4.061	***	−0.329
AP	←	MD	0.144	0.036	4.044	***	0.342
SE	←	MD	0.299	0.207	1.439	0.150	0.128
SE	←	VO_2_max	0.228	0.100	2.284	0.022	0.204
AP	←	VO_2_max	0.030	0.017	1.729	0.084	0.146
PA	↔	ST	−0.066	0.102	−0.648	0.517	−0.058

Note: regression weight (RW), standardised regression weight (SRW), standard error (SE), critical ratio (CR), maximal oxygen uptake (VO_2_max), physical activity (PA), screen time (ST), Mediterranean diet (MD), self-esteem (SE), academic performance (AP). *** *p* < 0.005.

**Table 5:** Regression weights pertaining to the SEM for participants attending state schools.

Association between Variables			RW				SRW
			Estimation	SE	CR	*p*	Estimation
VO_2_max	←	PA	1.956	0.713	2.745	0.006	0.247
VO_2_max	←	ST	−0.804	0.400	−2.010	0.044	−0.181
MD	←	PA	1.198	0.257	4.657	***	0.405
MD	←	VO_2_max	−0.030	0.033	−0.904	0.366	−0.080
MD	←	ST	−0.351	0.142	−2.467	0.014	−0.211
AP	←	MD	0.067	0.045	1.488	0.137	0.139
SE	←	MD	0.628	0.243	2.585	0.010	0.231
SE	←	VO_2_max	0.212	0.091	2.346	0.019	0.209
AP	←	VO_2_max	0.001	0.017	0.052	0.958	0.005
PA	↔	ST	−0.190	0.121	−1.564	0.118	−0.149

Note: regression weight (RW), standardised regression weight (SRW), standard error (SE), critical ratio (CR), maximal oxygen uptake (VO_2_max), physical activity (PA), screen time (ST), Mediterranean diet (MD), self-esteem (SE), academic performance (AP). *** *p* < 0.005.

**Table 6:** Regression weights pertaining to the SEM for participants attending mixed funding schools.

Association between Variables			RW				SRW
			Estimation	SE	CR	*p*	Estimation
VO_2_max	←	PA	2.636	0.571	4.614	***	0.348
VO_2_max	←	ST	−0.503	0.322	−1.560	0.119	−0.118
MD	←	PA	1.479	0.260	5.693	***	0.435
MD	←	VO_2_max	−0.032	0.034	−0.929	0.353	−0.071
MD	←	ST	−0.373	0.138	−2.697	0.007	−0.195
AP	←	MD	0.130	0.032	4.073	***	0.310
SE	←	MD	0.322	0.149	2.157	0.031	0.171
SE	←	VO_2_max	0.107	0.067	1.596	0.110	0.126
AP	←	VO_2_max	0.023	0.014	1.636	0.102	0.125
PA	↔	ST	−0.203	0.098	−2.072	0.038	−0.169

Note: regression weight (RW), standardised regression weight (SRW), standard error (SE), critical ratio (CR), maximal oxygen uptake (VO_2_max), physical activity (PA), screen time (ST), Mediterranean diet (MD), self-esteem (SE), academic performance (AP). *** *p* < 0.005.

## Data Availability

The data presented in this study are available on request from the corresponding author. The data are not publicly available due to the database is not published in any public repository.
